# Supplementary material for: Low growth resilience to drought is related to future mortality risk in trees
Source: Nat Commun. 2020 Jan 28;11:545. doi: 10.1038/s41467-020-14300-5 (PMC6987235; doi:10.1038/s41467-020-14300-5)
Supplement: Supplementary file 1 — Supplementary Information [file 41467_2020_14300_MOESM1_ESM.pdf]

Supplementary Information

Low growth resilience to drought is related to future mortality risk in trees

DeSoto *et al.*

Supplementary Tables

Table 1 Results of Type III test of the full linear mixed models of resilience, resistance and recovery computed for tree-ring width (TRW). Log-transformed resilience, resistance and recovery were considered as the response variable assuming a Gaussian error distribution with an identity link. Models include status (surviving vs. now-dead), taxonomic group (angiosperm vs. gymnosperm), diameter at breast height (DBH_i), time period between drought event and last year recorded in the individual ring-width series (Δtime), Standardised Precipitation Evapotranspiration Index during the drought event ($SPEI_i$), SPEI difference around the drought event (SPEIdiff), average ratio between precipitation and potential evapotranspiration (aridity index) for the period 1970-2000, soil characteristics (soil) and the interactions between status and all other fixed effects. The random part of the model includes the interaction of sites nested within species nested within genus. Data represent the degrees of freedom (df num, df den), the *F*-statistic and χ^2-statistic with the associated *P*-value of significance (bold type for significant effects, $P < 0.05$).

	**Resilience**				**Resistance**				**Recovery**			
**Fixed effects**	**df num**	**df den**	***F***	***P***	**df num**	**df den**	***F***	***P***	**df num**	**df den**	***F***	***P***
intercept	1	57.6	1.70	0.198	**1**	**42.7**	**9.07**	**0.004**	**1**	**53.1**	**5.67**	**0.021**
status	1	2818.7	2.87	0.09	1	3030.2	0.07	0.786	**1**	**3184.5**	**5.02**	**0.025**
group	1	9.8	0.06	0.813	1	11.6	0.14	0.719	1	8.6	0.37	0.559
DBH_i	**1**	**3008.3**	**4.76**	**0.029**	1	3149.6	0.48	0.488	1	3236.6	1.27	0.26
Δtime	**1**	**1784.7**	**4.99**	**0.026**	1	2599	3.24	0.072	1	2801.4	0.59	0.443
$SPEI_i$	1	79.1	0.43	0.512	--	--	--	--	--	--	--	--
SPEIdiff	1	55.8	0.14	0.711	1	93.3	0.58	0.450	1	93.2	0.98	0.325
aridity	1	38.8	0.01	0.928	1	27.2	0.02	0.900	1	65.6	0.19	0.663
soil fertility	1	36.4	0.00	0.972	**1**	**35.5**	**6.36**	**0.016**	1	48.1	3.11	0.084
status × group	1	3136.6	0.32	0.574	**1**	**3122**	**3.76**	**0.053**	**1**	**3186.9**	**6.10**	**0.014**
status × DBH_i	1	3142.2	2.62	0.105	1	3127.4	0.06	0.811	1	3196.4	0.97	0.324
status × Δtime	**1**	**3019.1**	**4.16**	**0.042**	1	3095.1	0.00	0.989	**1**	**3224.1**	**3.88**	**0.049**
status × $SPEI_i$	1	3182.3	3.70	0.055	--	--	--	--	--	--	--	--
status × SPEIdiff	1	3188.4	1.71	0.191	1	3160.8	0.78	0.376	1	3228.7	0.18	0.674
status × aridity	**1**	**3141.8**	**12.74**	**<0.001**	**1**	**3142.2**	**4.02**	**0.045**	1	3217.5	0.97	0.324
status × soil fertility	**1**	**2956.6**	**20.76**	**<0.001**	1	3085.2	0.31	0.576	**1**	**3215.3**	**10.41**	**0.001**
**Random effects**		**df**	**χ^2**	***P***		**df**	**χ^2**	***P***		**df**	**χ^2**	***P***
genus (species (site))		3	707.52	< 0.001		3	1113.70	< 0.001		3	942.62	< 0.001
No. of trees / sites	3207 / 104				3211 / 104				3276 / 104			
No. of species / genus	21/10				21/10				21/10			

Table 2 Differences in the performance of the full and reduced models fitted for each response variable (resilience, resistance and recovery) computed for tree-ring width (TRW) data. Reduced models including status (now-dead or surviving, reduced) or not (reduced - status) as fixed effect are tested for comparison. Full models (full) are also compared with reduced models, full models including mortality (full + mortality) or mortality instead of taxonomic group (full - group + mortality), and including the interaction between taxonomic group and aridity index (full + group × aridity) as fixed effects. Models selection was done following the Akaike Information Criterion (AIC). Data include, df, degrees of freedom; ΔAIC, increment on AIC values with respect to that of the model selected with lowest AIC, and statistical tests (χ^2, df, *P*) for the differences between models. Larger differences (ΔAIC) > 2.0 are shown in bold. See Table 1, and Supplementary Tables 1, 6, 7, 8 and 9 for models descriptions.

	**model**	**df**	**AIC**	**ΔAIC**	**χ2**	**df**	***P***
**Resilience**	reduced	13	-3679.2				
	reduced - status	**9**	**-3657.0**	**22.2**	**30.21**	**4**	**< 0.001**
	full	20	-3675.0	4.2	9.84	7	0.200
	full + mortality	32	-3662.2	12.8	11.15	12	0.516
	full - group + mortality	30	-3665.1	9.9	10.05	10	0.437
	Full + group × aridity	21	-3673.1	1.9	0.07	1	0.792
**Resistance**	reduced	11	-2877.9				
	reduced - status	**8**	**-2859.9**	**18.0**	**24.03**	**3**	**< 0.001**
	full	18	-2459.7	418.2	8.30	7	0.307*
	full + mortality	30	-2453.9	5.8	18.23	12	0.109
	full - group + mortality	28	-2449.2	10.5	9.46	10	0.489
	Full + group × aridity	19	-2457.8	1.9	0.05	1	0.820
**Recovery**	reduced	10	-2715.4				
	reduced - status	**7**	**-2710.8**	**4.6**	**10.64**	**3**	**0.014**
	reduced + mortality	22	-2700.3	15.1	8.88	12	0.713
	full	18	-2290.5	424.9	14.83	8	0.063*
	full - group + mortality	28	-2279.4	11.1	8.93	10	0.539
	Full + group × aridity	19	-2288.5	2.0	0.06	1	0.811

*models were compared using the same sample size

Table 3 Results of Type III test of the full linear mixed models of resilience, resistance and recovery computed for basal area increment (BAI). Log-transformed resilience, resistance and recovery were considered as the response variable assuming a Gaussian error distribution with an identity link. Models include status (surviving vs. now-dead), taxonomic group (angiosperm vs. gymnosperm), diameter at breast height (DBH_i), time period between drought event and last year recorded in the individual ring-width series (Δtime), Standardised Precipitation Evapotranspiration Index during the drought event ($SPEI_i$), SPEI difference around the drought event (SPEIdiff), average ratio between precipitation with potential evapotranspiration (aridity index) for the period 1970-2000, soil characteristics (soil) and the interactions between status and all other fixed effects. The random part of the model includes the interaction of sites nested within species nested within genus. Data represent the degrees of freedom (df num, df den), the *F*-statistic and χ^2-statistic with the associated *P*-value of significance (bold type for significant effects, $P < 0.05$).

	**Resilience**				**Resistance**				**Recovery**			
**Fixed effects**	**df num**	**df den**	***F***	***P***	**df num**	**df den**	***F***	***P***	**df num**	**df den**	***F***	***P***
intercept	1	54.6	1.08	0.304	**1**	**40.0**	**2.78**	**0.103**	**1**	**40.8**	**5.49**	**0.024**
status	1	2477.3	1.95	0.163	1	2688.9	0.57	0.451	1	2722.2	3.55	0.060
group	1	7.2	2.06	0.193	1	6.8	0.01	0.942	1	6.7	0.51	0.501
DBH_i	**1**	**2610.7**	**23.16**	**<0.001**	1	2693.6	2.13	0.144	1	2737.7	0.82	0.366
Δtime	1	1985.9	3.41	0.065	1	2655.4	2.09	0.148	1	2607.3	0.03	0.854
$SPEI_i$	1	105.1	0.02	0.884	--	--	--	--	--	--	--	--
SPEIdiff	1	94.1	2.71	0.103	1	89.0	0.29	0.589	1	93.1	2.38	0.126
aridity	1	50.0	0.16	0.695	1	66.4	0.52	0.474	1	66.6	0.54	0.463
soil	1	31.2	0.34	0.566	1	35.0	1.01	0.323	1	37.1	2.25	0.142
status × group	1	2660.3	0.89	0.346	**1**	**2645.3**	**5.46**	**0.019**	**1**	**2686.5**	**8.41**	**0.004**
status × DBH_i	1	2666.1	2.91	0.088	1	2649.0	0.27	0.600	1	2693.2	0.16	0.685
status × Δtime	1	2579.6	1.24	0.266	1	2687.4	0.26	0.610	1	2725.6	2.10	0.148
status × $SPEI_i$	1	2691.3	1.43	0.232	--	--	--	--	--	--	--	--
status × SPEIdiff	**1**	**2700.7**	**5.50**	**0.019**	1	2670.0	0.02	0.893	1	2696.8	0.10	0.747
status × aridity	**1**	**2680.4**	**12.99**	**<0.001**	1	2667.1	0.90	0.344	1	2713.4	0.31	0.575
status × soil	**1**	**2474.5**	**14.81**	**<0.001**	1	2684.1	0.08	0.782	**1**	**2719.7**	**4.93**	**0.027**
**Random effects**		**df**	**χ^2**	***P***		**df**	**χ^2**	***P***		**df**	**χ^2**	***P***
genus (species (site))		3	638.18	< 0.001		3	678.75	< 0.001		3	525.71	< 0.001
No. of trees / sites	2723 / 86				2727 / 86				2767 / 86			
No. of species / genus	19/9				19/9				19/9			

Table 4 Summary of the fitted linear mixed model of resilience, resistance and recovery computed for basal area increment (BAI). The response variables are log-transformed resistance, recovery and resilience, assuming a Gaussian error distribution with an identity link. The fixed part of the model included status (now-dead or surviving), taxonomic group (angiosperm or gymnosperm), diameter at breast height (DBHi, cm), time period between drought event and last year recorded in each individual tree ring-width series (Δtime, years), average ratio between precipitation and potential evapotranspiration (aridity) for the period 1970-2000, a measure of soil fertility, and interactions between status and other fixed effects. The random part of the model included site nested within species nested within genus. The intercept corresponds to the reference status (now-dead) and taxonomic group (angiosperms). This summary corresponds to the reduced (the full model is presented in Supplementary Table 3, for models selection see Supplementary Table 5). Values represent the standardised estimates of regression coefficients (std. *ß*), 95 % confidence intervals (CI), the *t*-statistic or χ^2-statistic and the associated P-value of significance (bold type for significant effects, P < 0.05). Estimates of regression coefficients for the intercept were not standardised. The signs indicate the direction of the effects. R^2m, marginal R^2; R^2c, conditional R^2; ΔAIC, increment on AIC values with respect to that of the model without status (Supplementary Table 5).

	Resilience					Resistance					Recovery				
**Fixed Effects**	***std. ß***	**CI**	**df**	***t***	***P***	***std. ß***	**CI**	**df**	***t***	***P***	***std. ß***	**CI**	**df**	***t***	***P***
(Intercept)	0.049	-0.007,0.106	25.7	1.63	0.115	-0.169	-0.400,0.063	29.4	-1.43	0.164	0.193	-0.005,0.391	29.4	1.86	0.073
surviving	-0.032	-0.064,0.001	2688.8	-1.89	0.059	**0.068**	**0.023,0.112**	**2636.4**	**2.98**	**0.003**	**-0.055**	**-0.103,-0.007**	**2676.6**	**-2.24**	**0.025**
gymnosperms	--	--	--	--	--	-0.049	-0.324,0.225	25.0	-0.35	0.732	-0.047	-0.280,0.185	22.0	-0.38	0.704
DBH_i	**-0.002**	**-0.003,-0.002**	**2305.1**	**-7.74**	**<0.001**	--	--	--	--	--	--	--	--	--	--
SPEIdiff	-0.041	-0.083,-0.001	110.0	-1.94	0.055	--	--	--	--	--	--	--	--	--	--
aridity	-0.005	-0.078,0.068	22.5	-0.13	0.899	--	--	--	--	--	--	--	--	--	--
soil fertility	-0.002	-0.013,0.009	15.3	-0.34	0.735	--	--	--	--	--	-0.037	-0.077,0.002	35.1	-1.86	0.072
surviving × gymnosperms	--	--	--	--	--	**-0.058**	**-0.111,-0.005**	**2639.5**	**-2.16**	**0.031**	**0.075**	**0.020,0.129**	**2679.5**	**2.69**	**0.007**
surviving × SPEIdiff	**0.045**	**0.020,0.069**	**2693.7**	**3.54**	**<0.001**	--	--	--	--	--	--	--	--	--	--
surviving × aridity	**0.060**	**0.023,0.096**	**2680.8**	**3.17**	**0.002**	--	--	--	--	--	--	--	--	--	--
surviving × soil fertility	**-0.009**	**-0.015,-0.003**	**2668.7**	**-2.91**	**0.004**	--	--	--	--	--	-0.011	-0.023,0.000	2682.8	-1.90	0.057
**Random effects**			***df***	***χ^2***	***P***			***df***	***χ^2***	***P***			***df***	***χ^2***	***P***
genus (species (site))			3	641.69	<0.001			3	879.74	<0.001			3	535.13	<0.001
No. of trees / sites	2723/86					2727/86					2767/86				
No. of species / genus	19/9					19/9					19/9				
R^2m / R^2c	0.06/0.32					0.01/0.60					0.04/0.55				
ΔAIC	38.8					5.3					3.8				

* The low marginal R^2 explained by the fixed effects of the reduced models might be a consequence of data heterogeneity, with high variation within species and sites (Cailleret et al., 2017). Nevertheless, differences between status were detected, and smaller AICs and larger differences (ΔAIC) > 2.0 related to models without status indicate that models including status showed higher explanatory power (Maestre et al., 2012).

Table 5 Difference in the performance of the full and reduced models fitted for each response variable (resilience, resistance and recovery) computed for basal area increment (BAI) data. Reduced models including status (now-dead or surviving, reduced) or not (reduce - status) as fixed effect are tested for comparison. Full models (full) are also compared with reduced models. Models selection was done following the Akaike Information Criterion (AIC). Data include, df, degrees of freedom; ΔAIC, increment on AIC values with respect to that of the model selected with lowest AIC and statistical tests (χ^2, df, *P*) for the differences between models. Larger differences (ΔAIC) > 2.0 are shown in bold. See Supplementary Tables 3 and 4 for models descriptions.

BAI	model	df	AIC	ΔAIC	$\chi 2$	df	*P*
**Resilience**	reduced	13	-2759.4				
	reduced - status	**9**	-2720.6	**38.8**	**46.80**	**4**	**< 0.001**
	full	20	-2763.9	-4.5	18.55	7	0.378
**Resistance**	reduced	8	1139.7				
	reduced - status	**6**	**1145.0**	**5.3**	9.33	**2**	**0.009**
	full	18	**1146.0**	**6.3**	13.68	8	0.188*
**Recovery**	reduced	10	1280.1				
	reduced - status	**7**	**1283.9**	**3.8**	**9.79**	**3**	**0.020**
	full	18	1287.4	7.3	8.75	8	0.364*

*models were compared using the same sample size

Table 6 Results of Type III test of the full linear mixed models of resilience, resistance and recovery computed for tree-ring width (TRW) considering the effects of any additional sources of mortality. Log-transformed resilience, resistance and recovery were considered as the response variable assuming a Gaussian error distribution with an identity link. Models include status (surviving vs. now-dead), taxonomic group (angiosperm vs. gymnosperm), additional mortality source (see Supplementary Data 1), diameter at breast height (DBH_i), Standardised Precipitation Evapotranspiration Index during the drought event ($SPEI_i$), SPEI difference around the drought event (SPEIdiff), time period between drought event and last year recorded in the individual ring-width series (Δtime), average ratio between precipitation and potential evapotranspiration (aridity index) for the period 1970-2000, soil characteristics (soil), and the interactions between status and all other fixed effects. The random part of the model includes the interaction of sites nested within species nested within genus. Data represent the degrees of freedom (df num, df den), the F-statistic with the associated P-value of significance (bold type for significant effects, $P < 0.05$).

	**Resilience**				**Resistance**				**Recovery**			
**Fixed effects**	**df num**	**df den**	***F***	***P***	**df num**	**df den**	***F***	***P***	**df num**	**df den**	***F***	***P***
intercept	1	48.8	0.74	0.393	1	32.1	6.52	0.016	**1**	**38.4**	**5.31**	0.027
status	1	2997.6	3.11	0.078	1	3114.3	0.33	0.563	**1**	**3218.0**	**7.12**	**0.008**
group	1	20.6	0.81	0.378	1	22.9	0.02	0.900	1	14.4	0.25	0.625
DBH_i	**1**	**2906.9**	**4.37**	**0.037**	1	3113.5	0.11	0.743	1	3205.4	1.54	0.215
Δtime	**1**	**2517.9**	**6.19**	**0.013**	1	3056.3	4.09	0.043	1	3049.6	0.32	0.570
additional mortality	6	25.8	0.68	0.667	6	16.5	0.42	0.856	6	27.1	0.67	0.671
$SPEI_i$	1	123.2	0.35	0.553	--	--	--	--	--	--	--	--
SPEIdiff	1	88.9	0.02	0.879	1	106.4	0.74	0.393	1	112.9	2.24	0.137
aridity	1	41.0	0.04	0.839	1	26.6	0.02	0.898	1	76.8	0.00	0.974
soil fertility	1	32.0	0.06	0.812	1	28.3	3.80	0.061	1	45.1	2.69	0.108
status × group	1	3136.7	0.40	0.527	**1**	**3123.1**	**7.85**	**0.005**	**1**	**3190.8**	**11.59**	**0.001**
status × DBH_i	1	3132.1	2.72	0.099	1	3114.3	0.48	0.487	1	3183.9	0.59	0.441
status × Δtime	1	2859.8	1.86	0.173	1	3052.9	0.02	0.893	1	3205.5	1.51	0.219
status × additional mortality	6	3138.9	0.54	0.779	6	3129.8	2.03	0.058	**6**	**3196.5**	**2.62**	**0.015**
status × $SPEI_i$	1	3175.4	1.31	0.252	--	--	--	--	--	--	--	--
status × SPEIdiff	1	3174.9	2.79	0.095	**1**	**3167.4**	**4.41**	**0.036**	**1**	**3221.3**	**5.61**	**0.018**
status × aridity	**1**	**3147.2**	**7.92**	**0.005**	**1**	**3167.2**	**13.45**	**< 0.001**	1	3232.5	2.44	0.118
status × soil fertility	**1**	**2961.5**	**17.02**	**< 0.001**	1	3120.5	3.15	0.076	1	3220.4	1.89	0.169
No. of trees / sites	3207 / 104				3211 / 104				3276 / 104			
No. of species / genus	21/10				21/10				21/10			

Table 7 Results of Type III test of the full linear mixed models of resilience, resistance and recovery computed for tree-ring width (TRW) considering the effects of any additional sources of mortality and not including taxonomic group. Log-transformed resilience, resistance and recovery were considered as the response variable assuming a Gaussian error distribution with an identity link. Models include status (surviving vs. now-dead), additional mortality source (see Supplementary Data 1), diameter at breast height (DBH_i), Standardised Precipitation Evapotranspiration Index during the drought event ($SPEI_i$), SPEI difference around the drought event (SPEIdiff), time period between drought event and last year recorded in the individual ring-width series (Δtime), average ratio between precipitation and potential evapotranspiration (aridity index) for the period 1970-2000, soil characteristics (soil), and the interactions between status and all other fixed effects. The random part of the model includes the interaction of sites nested within species nested within genus. Data represent the degrees of freedom (df num, df den), the *F*-statistic with the associated *P*-value of significance (bold type for significant effects, $P < 0.05$).

	Resilience				Resistance				Recovery			
**Fixed effects**	**df num**	**df den**	***F***	***P***	**df num**	**df den**	***F***	***P***	**df num**	**df den**	***F***	***P***
intercept	**1**	**45.7**	**4.35**	**0.043**	**1**	**17.9**	**13.16**	**0.002**	**1**	**31.0**	**6.85**	**0.014**
status	1	2648.2	2.57	0.109	1	2978.0	0.16	0.686	1	3151.5	2.46	0.117
DBH_i	**1**	**2877.5**	**4.07**	**0.044**	1	3091.3	0.47	0.492	1	3202.7	0.81	0.367
Δtime	**1**	**2535**	**6.24**	**0.013**	1	3044.4	3.57	0.059	1	3058.0	0.53	0.465
additional mortality	6	23.4	0.56	0.757	6	10.6	0.39	0.871	6	26.9	0.71	0.647
$SPEI_i$	1	114.8	0.92	0.339	--	--	--	--	--	--	--	--
SPEIdiff	1	90.1	0.01	0.937	1	98.6	0.52	0.471	1	111.4	1.54	0.217
aridity	1	39.1	0.05	0.832	1	15.2	0.08	0.785	1	79.6	0.02	0.879
soil fertility	1	32.0	0.04	0.838	**1**	**23.4**	**5.13**	**0.033**	1	47.2	2.53	0.118
status × DBH_i	1	3137.1	2.99	0.084	1	3117.8	0.06	0.800	1	3188.2	1.46	0.228
status × Δtime	1	2882.3	2.04	0.154	1	3058.8	0.06	0.800	1	3212.5	3.26	0.071
status × additional mortality	6	3140.8	0.52	0.794	6	3130.7	1.39	0.215	6	3196.7	1.73	0.109
status × $SPEI_i$	1	3163.0	2.06	0.152	--	--	--	--	--	--	--	--
status × SPEIdiff	1	3176.6	2.57	0.109	1	3171.6	2.54	0.111	1	3230.4	2.14	0.143
status × aridity	**1**	**3161.4**	**8.73**	**0.003**	**1**	**3171.9**	**11.17**	**0.001**	1	3235.3	1.09	0.297
status × soil fertility	**1**	**3007.5**	**18.41**	**< 0.001**	1	3132.3	2.09	0.148	**1**	**3226.3**	**4.13**	**0.042**
No. of trees / sites	3207 / 104				3211 / 104				3276 / 104			
No. of species / genus	21/10				21/10				21/10			

Table 8 Summary of the fitted linear mixed model of recovery computed for tree-ring width (TRW) considering the effects of any additional sources of mortality. The response variable is the log-transformed recovery assuming a Gaussian error distribution with an identity link. The fixed part of the model included status (now-dead or surviving), taxonomic group (angiosperm or gymnosperm), additional mortality source (codes in Supplementary Data 1), SPEI difference during the period of drought event (SPEIdiff), and interactions between status and the other fixed effects. The intercept corresponds to the reference status (now-dead) and taxonomic group (angiosperms). This summary corresponds to the reduced model that includes the significant effects of the full model (presented in Supplementary Table 1, for models selection see Supplementary Table 2). Values represent the standardised estimates of regression coefficients (β), the 95 % confident intervals (CI), the degrees of freedom (df), the t-statistic and the associated P-value of significance (bold type for significant effects, $P < 0.05$).

	Recovery				
**Fixed Effects**	***std. β***	**CI**	**df**	***t***	***P***
(Intercept)	**0.178**	**0.059,0.290**	**45.4**	**2.83**	**0.007**
surviving	**-0.089**	**-0.136,-0.042**	**3622.7**	**-3.67**	**<0.001**
gymnosperms	-0.031	-0.147,0.084	26.6	-0.48	0.638
mortality Bb	0.049	-0.031,0.139	95.6	1.09	0.278
mortality Bb_F	-0.031	-0.314,0.254	42.7	-0.20	0.841
mortality C	-0.090	-0.235,0.067	84.9	-1.11	0.270
mortality F	-0.026	-0.140,0.089	76.0	-0.42	0.678
mortality M	-0.108	-0.242,0.044	108.6	-1.46	0.147
mortality Wb	0.059	-0.147,0.263	12.4	0.50	0.623
SPEIdiff	-0.015	-0.040,0.019	124.5	-1.05	0.294
surviving × gymnosperms	**0.082**	**0.041,0.123**	**3613.7**	**3.93**	**<0.001**
surviving × mortality Bb	0.003	-0.030,0.038	3627.1	0.20	0.844
surviving × mortality Bb_F	-0.002	-0.097,0.093	3602.3	-0.04	0.967
surviving × mortality C	-0.026	-0.087,0.034	3618.6	-0.85	0.396
surviving × mortality F	**0.050**	**0.007,0.094**	**3637.4**	**2.26**	**0.024**
surviving × mortality M	0.017	-0.033,0.066	3615.7	0.66	0.511
surviving × mortality Wb	**0.075**	**0.027,0.122**	**3607.4**	**3.08**	**0.002**
surviving × SPEIdiff	**0.014**	**0.001,0.027**	**3643.0**	**2.16**	**0.031**

Table 9 Results of Type III test of the full linear mixed models of resilience, resistance and recovery computed for tree-ring width (TRW) considering the effects of the interaction between aridity index and taxonomic group. Log-transformed resilience, resistance and recovery were considered as the response variable assuming a Gaussian error distribution with an identity link. Models include status (surviving vs. now-dead), taxonomic group (angiosperm vs. gymnosperm), diameter at breast height (DBH_i), Standardised Precipitation Evapotranspiration Index during the drought event ($SPEI_i$), SPEI difference around the drought event (SPEIdiff), time period between drought event and last year recorded in the individual ring-width series (Δtime), average ratio between precipitation and potential evapotranspiration (aridity index) for the period 1970-2000, soil characteristics (soil), the interactions between status and all other fixed effects, and the interaction between aridity index and taxonomic group. The random part of the model includes the interaction of sites nested within species nested within genus. Data represent the degrees of freedom (df num, df den), the *F*-statistic with the associated *P*-value of significance (bold type for significant effects, $P < 0.05$).

	Resilience				Resistance				Recovery			
**Fixed effects**	**df num**	**df den**	***F***	***P***	**df num**	**df den**	***F***	***P***	**df num**	**df den**	***F***	***P***
intercept	1	44.9	0.59	0.446	**1**	**51.3**	**4.08**	**0.049**	1	43	2.32	0.135
status	1	2820.3	2.91	0.088	1	3047.7	0.08	0.777	**1**	**3183.6**	**5.05**	**0.025**
group	1	34.4	0.02	0.876	1	42.6	0.12	0.729	1	34.1	0.13	0.717
DBH_i	**1**	**2983.6**	**4.74**	**0.029**	1	3141.6	0.49	0.484	1	3225.6	1.26	0.262
Δtime	**1**	**1845.6**	**5.04**	**0.025**	1	2681.2	3.17	0.075	1	2824.2	0.6	0.437
$SPEI_i$	1	79.6	0.39	0.535	--	--	--	--	--	--	--	--
SPEIdiff	1	55	0.13	0.715	1	96.6	0.56	0.457	1	93.2	0.98	0.326
aridity	1	33.8	0	0.991	1	41.2	0.03	0.855	1	39.7	0.09	0.769
soil fertility	1	35.4	0	0.961	**1**	**34.8**	**5.71**	**0.022**	1	46.4	2.97	0.091
status × group	1	3136.4	0.31	0.575	**1**	**3121**	**3.76**	**0.052**	**1**	**3186.5**	**6.11**	**0.014**
status × DBH_i	1	3138.7	2.61	0.106	1	3125.7	0.06	0.814	1	3194.6	0.98	0.323
status × Δtime	**1**	**3037.4**	**4.2**	**0.04**	1	3119	0	0.981	**1**	**3225.5**	**3.9**	**0.048**
status × $SPEI_i$	1	3183.5	3.68	0.055	--	--	--	--	--	--	--	--
status × SPEIdiff	1	3187.6	1.71	0.191	1	3160.4	0.8	0.372	1	3228	0.18	0.671
status × aridity	**1**	**3134.8**	**12.79**	**0.001**	**1**	**3140.6**	**3.99**	**0.046**	1	3214.1	0.98	0.322
status × soil fertility	**1**	**2970.9**	**20.83**	**<0.001**	1	3104.8	0.3	0.583	**1**	**3216.1**	**10.44**	**0.001**
group × aridity	1	32.8	0	0.963	1	38	0.05	0.827	1	38.8	0.01	0.909
No. of trees / sites	3207 / 104				3211 / 104				3276 / 104			
No. of species / genus	21/10				21/10				21/10			

Supplementary Figures

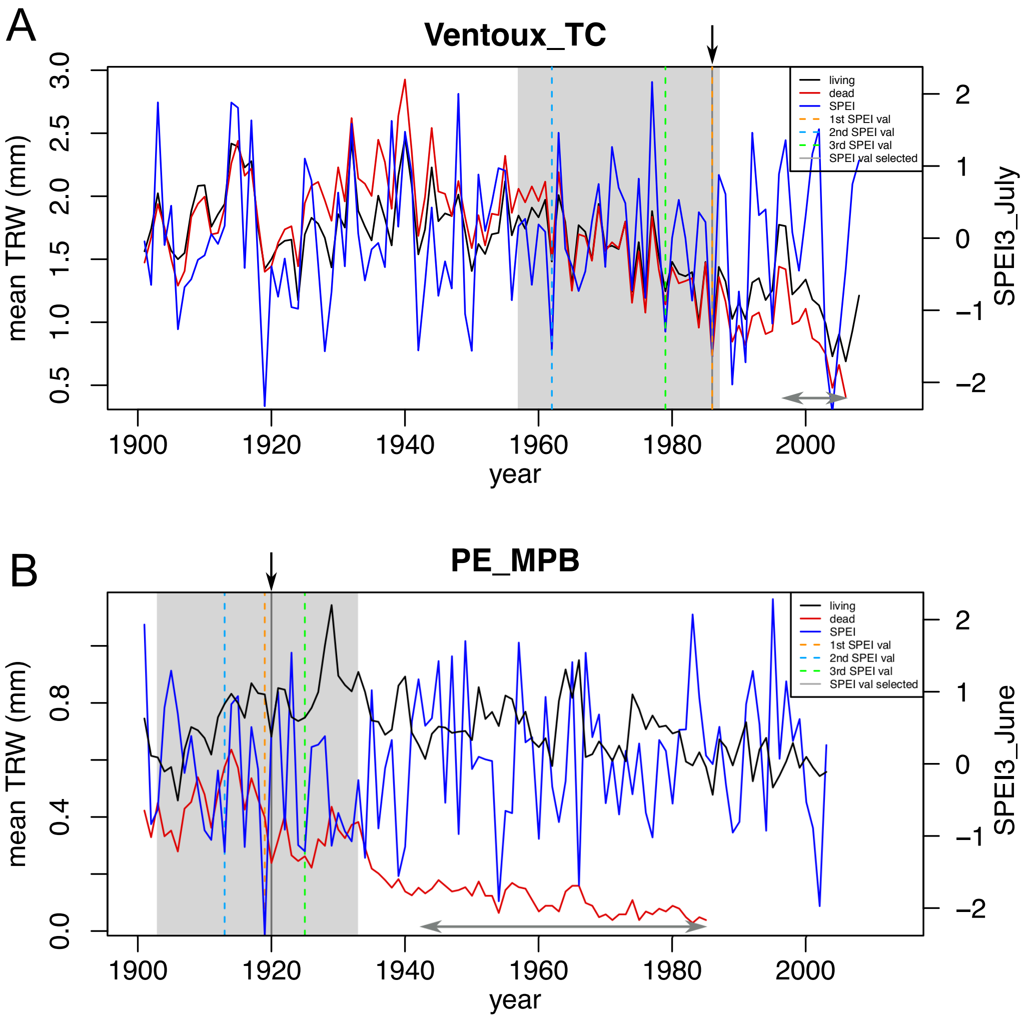

Fig. 1 Temporal patterns of tree growth and annual aridity showing extreme drought events. Mean tree-ring width for surviving (black line) and now-dead (red line) trees plotted over the Standardised Precipitation Evapotranspiration Index (SPEI, blue line) for two examples of drought event (black arrow) selection in sites that met the criteria explained in Methods, showing a response to drought **a** the same year (Ventoux_TC site, *Abies alba*), or **b** the year after (PE_MPB site, *Picea engelmannii*). The SPEI window is indicated with the month scale and the target month (e.g. SPEI3_July, SPEI for the 3-month scale for July, it considers the SPEI values from May to July in Northern Hemisphere). Minimum values of SPEI (1st SPEI val, orange dashed line; 2nd SPEI val, blue dashed line; and 3rd SPEI val, green dashed line) below the 10 % percentile of the SPEI distribution for each site are indicated for the common period (grey shadow). The period of tree death on each site is shown with grey arrows.

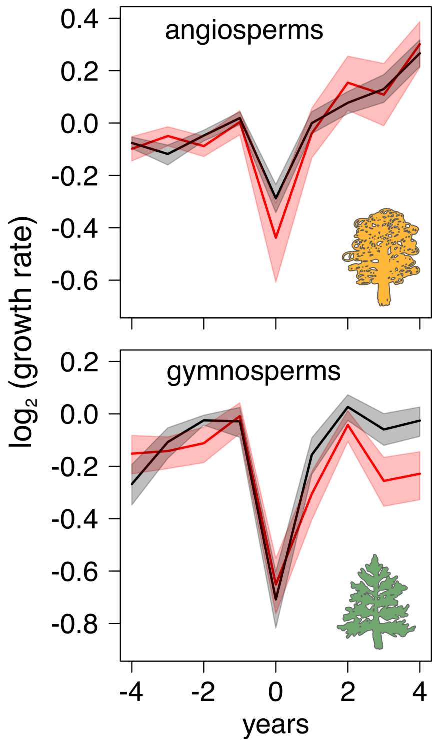

Fig. 2 Relative growth patterns before, during and after the drought event studied (year=0) for angiosperms and gymnosperms. Data are presented as the average of log-ratio between basal area increment (BAI) at a given year and the average growth for the four-year pre-drought period for surviving (black lines) and now-dead (red lines) trees. Shaded areas represent the 95% confidence intervals of the means from bootstrapping (1,000 resamplings). Source data are available in Digital.CSIC repository.

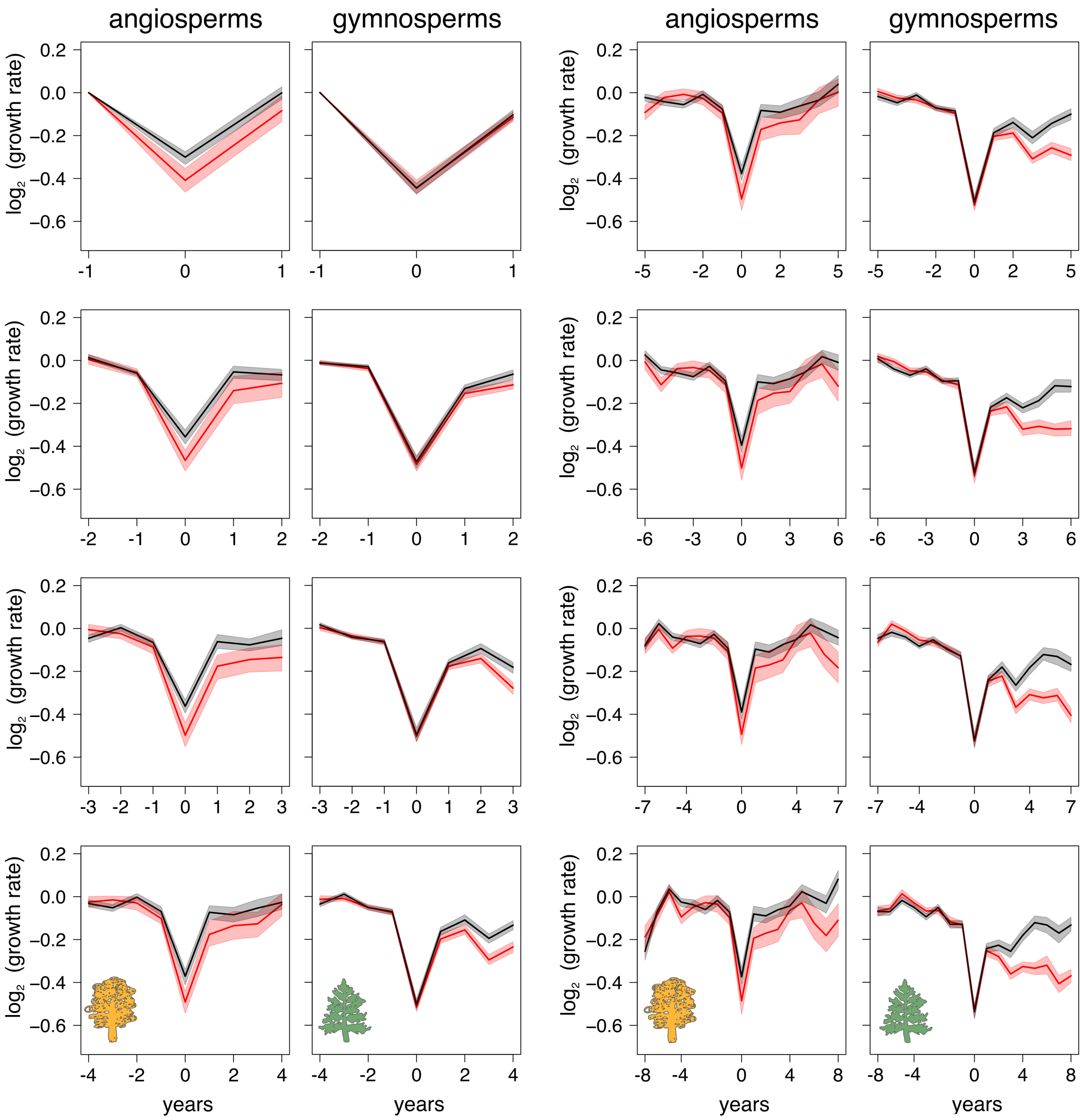

Fig. 3 Growth patterns before, during and after the drought event studied (year=0) for angiosperms and gymnosperms. Data are presented as the average of log-ratio between tree-ring width (TRW) at a given year and the average growth for the one to eight-year pre-drought period for surviving (black lines) and now-dead (red lines) trees. Shaded areas represent the 95% confidence intervals of the means from bootstrapping (100 resamplings). Source data are available in Digital.CSIC repository.

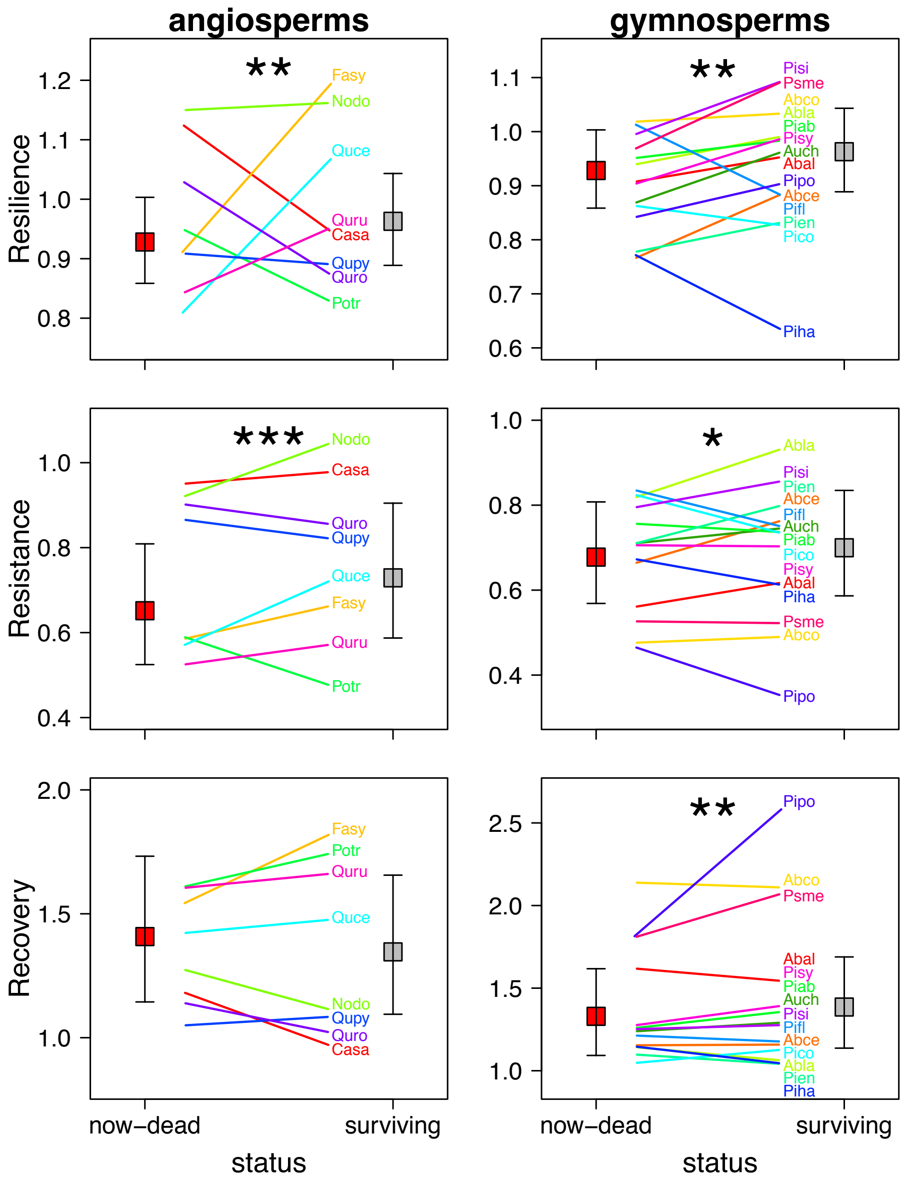

Fig. 4 Differences in resilience, resistance and recovery between now-dead and surviving trees as a function of taxonomic group (angiosperms vs. gymnosperms). The data are presented as model-adjusted back-transformed least-square means ± 95% confident intervals of surviving (grey squares) and now-dead (red squares) trees (Table 1). Lines connect mean resilience, resistance and recovery of surviving and now-dead for each studied species (Abal, *Abies alba*; Abce, *Abies cephalonica*; Abco, *Abies concolor*; Abla, *Abies lasiocarpa*; Auch, *Austrocedrus chilensis*; Casa, *Castanea sativa*; Fasy, *Fagus sylvatica*; Nodo, *Nothofagus dombeyi*; Piab, *Picea abies*; Pico, *Pinus contorta*; Pien, *Picea engelmannii*; Pifl, *Pinus flexilis*; Piha, *Pinus halepensis*; Pipo, *Pinus ponderosa*; Pisi, *Pinus sibirica*; Pisy, *Pinus sylvestris*; Potr, *Populus tremuloides*; Psme, *Pseudotsuga menziesii*; Quce, *Quercus cerris*; Qupy, *Quercus pyrenaica*; Quro, *Quercus robur*; Quru, *Quercus rubra*). Asterisks indicate significant pairwise differences in Least square-means between now-dead and surviving trees (LMM test: *, $P < 0.05$; **, $P < 0.01$; ***, $P < 0.001$). Source data are available in Digital.CSIC repository.

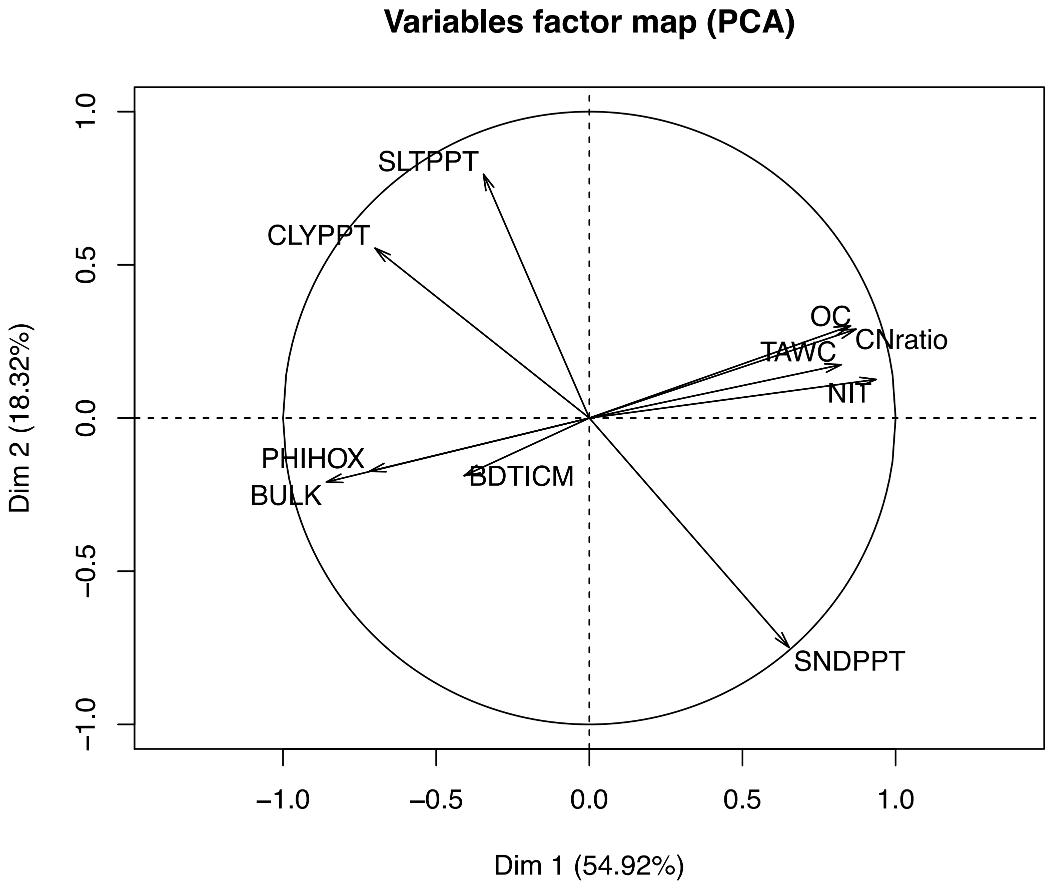

Fig. 5 First (Dim 1) and second (Dim2) principal components of soil characteristics in the studied sites. OC, organic carbon (g kg^{-1}); NIT, total nitrogen(g kg^{-1}); CNratio, carbon / nitrogen ratio; BULK, bulk density (kg dm^{-3}); TAWC, available water capacity (from -33 to -1500 kPa; cm m^{-1}); BDTICM, absolute depth to bed Rock (cm); PHIHOX, pH measured at 200cm; CLYPPT, clay content (%) measured at 60cm; SLTPPT, silt content (%) measured at 60cm; SNDPPT, sand content (%) measured at 60 cm. The percentage that explained each principal component is shown. Note, CNratio was calculated 'as is' from the measured data, not as the ratio of the derived values for C and N.

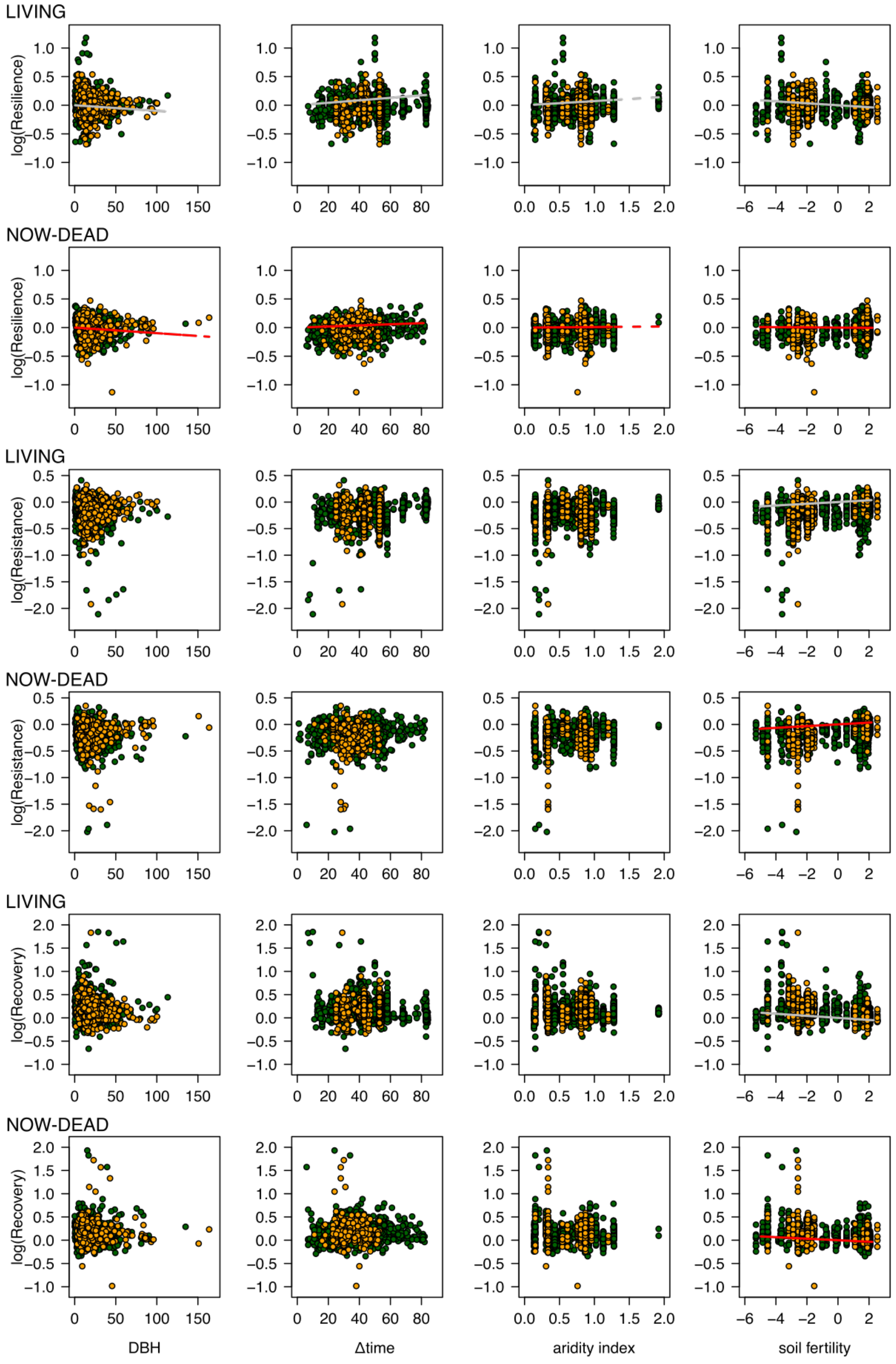

Fig. 6 Relationship between resilience, resistance or recovery and fixed effects. DBH_i, diameter at breast height; Δtime, time period between drought event and last year recorded in the individual ring-width series; aridity index, average ratio between precipitation and potential evapotranspiration for the period 1970-2000, or soil fertility are the analysed for surviving and now-dead trees of angiosperms (orange) and gymnosperms (green). Lines represent predicted values for the relationship between the predictor and log-transformed resilience, resistance and recovery indices computed for tree ring width (TRW) for surviving (grey) and now-dead (red) trees based on the linear mixed models in Table 1. Note, the aridity index indicates rainfall over potential vegetation water demand, and thus its value increases under more humid conditions, and decreases with more arid conditions. Source data are available in Digital.CSIC repository.

Supplementary References

References of the datasets already published that support the findings of this study.

1. Bigler C, Gričar J, Bugmann H, Čufar K (2004) Growth patterns as indicators of impending tree death in silver fir. Forest Ecology and Management, **199**, 183-190.
2. Cailleret M, Nourtier M, Amm A, Durand-Gillmann M, Davi H (2014) Drought-induced decline and mortality of silver fir differ among three sites in Southern France. Annals of Forest Science, **71**, 643-657.
3. Linares JC, Camarero JJ (2012) Growth patterns and sensitivity to climate predict silver fir decline in the Spanish Pyrenees. European Journal of Forest Research, **131**, 1001-1012.
4. Papadopoulos A, Raftoyannis Y, Pantera A (2007) Fir decline in Greece: a dendroclimatological approach. Proceedings of the 10th International Conference on Environmental Science and Technology. Kos Island, Greece.
5. Kane JM, Kolb TE (2014) Short-and long-term growth characteristics associated with tree mortality in southwestern mixed-conifer forests. Canadian Journal of Forest Research, **44**, 1227-1235.
6. Bigler C, Gavin DG, Gunning C, Veblen TT (2007) Drought induces lagged tree mortality in a subalpine forest in the Rocky Mountains. Oikos, **116**, 1983-1994.
7. Amoroso MM, Daniels LD, Larson BC (2012) Temporal patterns of radial growth in declining *Austrocedrus chilensis* forests in Northern Patagonia: the use of tree-rings as an indicator of forest decline. Forest Ecology and Management, **265**, 62-70.
8. Villalba R, Veblen TT (1998) Influences of large-scale climatic variability on episodic tree mortality in northern Patagonia. Ecology, **79***,* 2624-2640.
9. Waldboth M, Oberhuber W (2009) Synergistic effect of drought and chestnut blight (*Cryphonectria parasitica*) on growth decline of European chestnut (*Castanea sativa*). Forest Pathology, **39**, 43-55.
10. Gillner S, Rüger N, Roloff A, Berger U (2013) Low relative growth rates predict future mortality of common beech (*Fagus sylvatica* L.). Forest Ecology and Management, **302**, 372-378.
11. Suárez ML, Ghermandi L, Kitzberger T (2004) Factors predisposing episodic drought-induced tree mortality in *Nothofagus* – site, climatic sensitivity and growth trends. Journal of Ecology, **92**, 954-966.
12. Aakala T, Kuuluvainen T (2011) Summer droughts depress radial growth of *Picea abies* in pristine taiga of the Arkhangelsk province, northwestern Russia. Dendrochronologia, **29**, 67-75.
13. Mäkinen H, Nöjd P, Mielikäinen K (2001) Climatic signal in annual growth variation in damaged and healthy stands of Norway spruce [*Picea abies* (L.) Karst.] in southern Finland. Trees, **15**, 177-185.
14. Smith JM, Hart SJ, Chapman TB, Veblen TT, Schoennagel T (2012) Dendroecological reconstruction of 1980s mountain pine beetle outbreak in lodgepole pine forests in northwestern Colorado. Ecoscience, **19**, 113-126.
15. Dorman M, Perevolotsky A, Sarris D, Svoray T (2015) The effect of rainfall and competition intensity on forest response to drought: lessons learned from a dry extreme. Oecologia, **177**, 1025-1038.
16. Kane JM, Kolb TE (2010) Importance of resin ducts in reducing ponderosa pine mortality from bark beetle attack. Oecologia, **164**, 601-609.
17. Kharuk VI, Im ST, Oskorbin PA, Petrov IA, Ranson KJ (2013) Siberian pine decline and mortality in southern Siberian Mountains. Forest Ecology and Management, **310**, 312-320.
18. Bigler C, Bräker O, Bugmann H, Dobbertin M, Rigling A (2006) Drought as an inciting mortality factor in Scots pine stands of the Valais, Switzerland. Ecosystems, **9**, 330-343.
19. Gea-Izquierdo G, Viguera B, Cabrera, M, Cañellas I (2014) Drought induced decline could portend widespread pine mortality at the xeric ecotone in managed mediterranean pine-oak woodlands. Forest Ecology and Management, **320**, 70-82.

20. Hereş AM, Martínez-Vilalta J, López BC (2012) Growth patterns in relation to drought-induced mortality at two Scots pine (*Pinus sylvestris* L.) sites in NE Iberian Peninsula. Trees, **26**, 621-630.
21. Oberhuber W (2001) The role of climate in the mortality of Scots pine (*Pinus sylvestris* L.) exposed to soil dryness. Dendrochronologia, **19**, 45-55.
22. Sangüesa-Barreda G, Linares JC, Camarero JJ (2013) Drought and mistletoe reduce growth and water-use efficiency of Scots pine. Forest Ecology and Management, **296**, 64-73.
23. Stojanović D, Levanič T, Matović B, Bravo-Oviedo A (2015) Climate change impact on a mixed lowland oak stand in Serbia. Annals of Silvicultural Research, **39**, 94-99.
24. Levanič T, Čater M, McDowell NG (2011) Associations between growth, wood anatomy, carbon isotope discrimination and mortality in a *Quercus robur* forest. Tree Physiology, **31**, 298-308.
25. Tulik M (2014) The anatomical traits of trunk wood and their relevance to oak (*Quercus robur* L.) vitality. European Journal of Forest Research, **133**, 845-855.
26. Haavik LJ, Stahle DW, Stephen FM (2011) Temporal aspects of *Quercus rubra* decline and relationship to climate in the Ozark and Ouachita Mountains, Arkansas. Canadian Journal of Forest Research, **41**, 773-781.
