# Supplementary material for: Low growth resilience to drought is related to future mortality risk in trees
Source: Nat Commun. 2020 Jan 28;11:545. doi: 10.1038/s41467-020-14300-5 (PMC6987235; doi:10.1038/s41467-020-14300-5)
Supplement: Supplementary file 3 — Description of Additional Supplementary Files [file 41467_2020_14300_MOESM3_ESM.docx]

**Description of Additional Supplementary Files**

**File Name**: Supplementary Data 1
**Description:** Main characteristics of tree-ring datasets considered in the present study. Data include the taxonomical data (species, family and group), the site name, the geographic coordinates (latitude and longitude), the article where dataset was presented, the sources of mortality, the number of now-dead and surviving trees considered, the time span and diameter at breast height (DBH) range of the trees and the period between the first and last tree dying in the population. The additional sources of mortality (ASmort) were determined from field observations and the following codes were used: Bb, bark beetles; C, competition; F, fungi; M, mistletoe; Wb, wood-borers.

**File Name:** Supplementary Data 2
**Description:** Extreme drought events considered for the study. Data include the species, the site name, the SPEI window, the drought year and the corresponding value of SPEI, and Aridity Index. SPEI window is indicated with the month scale and the target month (e.g. SPEI22_08, SPEI for the 22-month scale for August, it considers the SPEI values from June of previous year to August in Northern Hemisphere).(*, indicates that drought year corresponds to the year after the drought event considered for the study because some tree species might delay its growth response to drought; e.g. AL_TCLA drought event occurred in 1919 with an intensity of SPEI = -1.92).
