# Supplementary material for: Low growth resilience to drought is related to future mortality risk in trees
Source: Nat Commun. 2020 Jan 28;11:545. doi: 10.1038/s41467-020-14300-5 (PMC6987235; doi:10.1038/s41467-020-14300-5)
Supplement: Supplementary file 6 — Reporting Summary [file 41467_2020_14300_MOESM6_ESM.pdf]

nature research

Corresponding author(s): Lucía DeSoto

Last updated by author(s): 26/11/19

Reporting Summary

Nature Research wishes to improve the reproducibility of the work that we publish. This form provides structure for consistency and transparency in reporting. For further information on Nature Research policies, see Authors & Referees and the Editorial Policy Checklist .

Statistics

For all statistical analyses, confirm that the following items are present in the figure legend, table legend, main text, or Methods section.

n/a	Confirmed	
☐	☒	The exact sample size (*n*) for each experimental group/condition, given as a discrete number and unit of measurement
☐	☒	A statement on whether measurements were taken from distinct samples or whether the same sample was measured repeatedly
☐	☒	The statistical test(s) used AND whether they are one- or two-sided<br>*Only common tests should be described solely by name; describe more complex techniques in the Methods section.*
☐	☒	A description of all covariates tested
☐	☒	A description of any assumptions or corrections, such as tests of normality and adjustment for multiple comparisons
☐	☒	A full description of the statistical parameters including central tendency (e.g. means) or other basic estimates (e.g. regression coefficient) AND variation (e.g. standard deviation) or associated estimates of uncertainty (e.g. confidence intervals)
☐	☒	For null hypothesis testing, the test statistic (e.g. *F*, *t*, *r*) with confidence intervals, effect sizes, degrees of freedom and *P* value noted<br>*Give P values as exact values whenever suitable.*
☒	☐	For Bayesian analysis, information on the choice of priors and Markov chain Monte Carlo settings
☐	☒	For hierarchical and complex designs, identification of the appropriate level for tests and full reporting of outcomes
☒	☐	Estimates of effect sizes (e.g. Cohen's *d*, Pearson's *r*), indicating how they were calculated

Our web collection on statistics for biologists contains articles on many of the points above.

Software and code

Policy information about availability of computer code

Data collection	No software were used to collect the data
Data analysis	We used the following R-packages “dplR”, “HighstatLib”, “lme4”, “car”, “emmeans” , “effects” and “lnme” in the R environment to analyse the data.

For manuscripts utilizing custom algorithms or software that are central to the research but not yet described in published literature, software must be made available to editors/reviewers. We strongly encourage code deposition in a community repository (e.g. GitHub). See the Nature Research guidelines for submitting code & software for further information.

Data

Policy information about availability of data

All manuscripts must include a data availability statement. This statement should provide the following information, where applicable:

- Accession codes, unique identifiers, or web links for publicly available datasets
- A list of figures that have associated raw data
- A description of any restrictions on data availability

The data that support the findings of this study are available from the Plant Trait database (TRY) , https://www.try-db.org/. The source data of figures 2 and 3 will be available soon in Digital.CSIC repository (https://digital.csic.es/?locale=en)

Field-specific reporting

Please select the one below that is the best fit for your research. If you are not sure, read the appropriate sections before making your selection.

☐ Life sciences ☐ Behavioural & social sciences ☒ Ecological, evolutionary & environmental sciences

Ecological, evolutionary & environmental sciences study design

All studies must disclose on these points even when the disclosure is negative.

Study description	We assessed whether trees that ultimately died during drought events (hereafter now-dead trees) were already less resilient to droughts that occurred decades before their death, relative to surviving trees from the same population. We also explored the differences between angiosperm and gymnosperm trees, due to their contrasting trait syndromes and drought response strategies. Finally, we assessed how the relationship between drought resilience and future mortality risk depends on the long-term water availability of each site (characterized by the aridity index calculated as the ratio between precipitation and potential evapotranspiration), on the intensity of the drought event under consideration and on soil properties. We quantify tree resilience to drought using the three indices proposed by Lloret et al. 2013 (Oikos 120:1909–1920): resistance (ratio between during- and pre-drought growth), recovery (ratio between post- and during-drought growth) and resilience s.str. (ratio between pre- and post-drought growth), considering four years before and after the drought event. We used linear mixed models (LMMs) to analyse resilience differences between coexisting now-dead and surviving trees. Resistance, recovery and resilience computed for tree-ring width (TRW) series were log-transformed to satisfy normality of the LMM residuals, and considered as the response variables assuming a Gaussian error distribution with an identity link. In the initial, full LMM, the fixed effects included were: (1) tree status (surviving vs. now-dead); (2) taxonomic group (angiosperms vs. gymnosperms); (3) diameter at breast height in the year of the drought event (DBHi, where i refers to the target drought event); (4) the relative intensity of the drought event, expressed as the Standardised Precipitation Evapotranspiration Index value during the drought event (SPEIi) and the SPEI difference corresponding to resistance, recovery and resilience periods; (5) the length of the time period between the drought event and the last year recorded in the ring-width series of each tree (Δtime), to control for temporal effects (mortality risk might be more related to a drought that occurred 10 years ago than 40 years ago); (6) the average ratio between precipitation and potential evapotranspiration as a measure of climatic aridity; (7) the first principal component of the soil PCA as a measure of soil fertility; and (8) all the interactions between tree status (surviving vs. now-dead) and each of the other fixed effects. In all LMMs, random effects were estimated for the intercept with site nested in species and species nested in genus as grouping factors. Then, we simplified each full LMM by removing the least significant terms until a minimum adequate model (in terms of AIC) was identified. Identical analyses were also performed for resilience indices based on basal area increment (BAI) instead of TRW. We included 118 sites with 2,456 and 1,454 co-occurring surviving and now-dead trees, respectively, of 22 species (8 angiosperms and 14 gymnosperms).
Research sample	We selected tree-ring width (TRW, mm) datasets from the pancontinental database compiled by Cailleret et al. 2017 (Global Change Biol 23:1675-1690), for which (i) both dying and surviving trees growing together at the same site were cored, (ii) all individual TRW series had been successfully cross-dated, and (iii) mortality was mainly induced by drought, solely or in combination with other factors, such as bark beetles, fungi or mistletoes. The database included 127 sites mostly located in the boreal, temperate and Mediterranean biomes of North America and Europe. We used two metrics of tree growth, TRW and basal area increment (BAI, mm2).
Sampling strategy	The data considered for this study was a subset of a larger database compiled already. The original database contains growth data from 58 published and unpublished research articles about tree mortality collected by Cailleret et al. 2017 (Global Change Biol 23:1675-1690).
Data collection	M.C., T.A., M.M.A., C.B., J.J.C., K.Č., G.G.-I., S.G., L.J.H., A.M.H., J.M.K., V.I.K., T.Ki., T.Kl., T.L., J.C.L., H.M., W.O., A.P., B.R., G.S.-B., D.B.S., M.L.S., R.V., and J.M.-V. collected the tree-ring data from published and unpublished research articles. The data collection was based upon work from COST Action FP1106 STReESS, supported by COST (European Cooperation in Science and Technology).
Timing and spatial scale	Our analysis is constrained by the available studies of tree mortality and radial growth, which are focused on extra-tropical forests (largely due to methodological constraints) and mainly in the Northern Hemisphere. Nevertheless, we included several populations of Austrocedrus and Nothofagus in the Southern Hemisphere and we covered vast areas in the Northern hemisphere between 31 and 63 degrees North and -112 and 93 East, and we included 22 species from 10 genera. Our database included tree population with a climatic water balance in terms of Aridity Index ranging between 0.14 and 1.92, from arid to humid climates.
Data exclusions	The exclusion criteria were pre-established. We selected only a single drought event for each site, because the frequency and intensity of drought events can differ among sites . The drought event was selected within a 30-year period following two steps. First, to discard exceptional long mortality periods, we excluded now-dead trees that died more than 50 years before the last death event recorded at a given site. Second, to avoid selecting drought events either too close or too distant in time to the mortality event, we limited the time period from 10 to 40 years before the first tree dying in each site. Within this period, we selected one drought event per site following two criteria: (1) SPEI < the 10th percentile of the site-specific SPEI distribution , and (2) abnormal low growth in the same year or in the year after (mean TRW of the site was reduced > 5% relative to the average TRW of the four previous years). Growth reductions the year after the drought were rare but were considered because, depending on drought timing and the species tolerance, some trees might show a delay in their growth response to drought . After discarding nine sites that did not meet the growth reduction criterion, we considered 118 sites with 2,456 and 1,454 co-occurring surviving and now-dead trees, respectively, of 22 species (8 angiosperms and 14 gymnosperms) for the subsequent analyses.
Reproducibility	No experimental design was carried out for this study.
Randomization	Samples (trees) from the database were allocated into their family, genera, species, site and status (surviving or now-dead).
Blinding	Blinding was not relevant in our study because we compiled tree growth data from every published and unpublished research articles about tree mortality that were available at 2016. Analyses were blinding by using Linear Mixed Models (but see data exclusions).

Did the study involve field work? ☐ Yes ☒ No

Reporting for specific materials, systems and methods

We require information from authors about some types of materials, experimental systems and methods used in many studies. Here, indicate whether each material, system or method listed is relevant to your study. If you are not sure if a list item applies to your research, read the appropriate section before selecting a response.

Materials & experimental systems

n/a	Involved in the study
☒	☐ Antibodies
☒	☐ Eukaryotic cell lines
☒	☐ Palaeontology
☒	☐ Animals and other organisms
☒	☐ Human research participants
☒	☐ Clinical data

Methods

n/a	Involved in the study
☒	☐ ChIP-seq
☒	☐ Flow cytometry
☒	☐ MRI-based neuroimaging
